# First reported cases of SARS-CoV-2 infection in pets in São Paulo, Brazil

**DOI:** 10.14202/vetworld.2022.2593-2596

**Published:** 2022-11-16

**Authors:** Rafael G. Agopian, Suellen C. G. da Luz, Alexandre G. B. Zebral, Giovanna F. de Sousa, Igor A. V. de Oliveira, Letícia S. Lima, Marcela A. Sechi, Mayara C. de Oliveira, Valéria F. Rudiniski, Daniel Friguglietti Brandespim, Otávio V. de Carvalho, Christina Pettan-Brewer, Andrea P. dos Santos, Louise B. Kmetiuk, Alexander Welker Biondo

**Affiliations:** 1 Department of Veterinary Medicine, Veterinary College, University of Santo Amaro (UNISA), São Paulo, Brazil; 2 Department of Medicine, University of Santo Amaro (UNISA), São Paulo, Brazil; 3 Department of Veterinary Medicine, College of Veterinary Medicine, Federal Rural University of Pernambuco, Recife, Pernambuco, Brazil; 4 TECSA Animal Laboratories, Belo Horizonte, Brazil; 5 Department of Comparative Medicine, School of Medicine, University of Washington, Seattle, Washington, USA; 6 Department of Comparative Pathobiology, College of Veterinary Medicine, Purdue University, West Lafayette, Indiana, USA; 7 Department of Veterinary Medicine, Federal University of Paraná, Curitiba, Paraná, Brazil

**Keywords:** novel coronavirus, One Health, veterinary medicine, zoonoses

## Abstract

**Background and Aim:**

The severe acute respiratory syndrome coronavirus 2 (SARS-CoV-2) is responsible for the global coronavirus pandemic (COVID-19) in humans in 2019. Although SARS-CoV-2 infection is primarily asymptomatic and transitory in companion animals, the role of these animals in the life cycle of the virus remains unclear. This study aimed to survey the first SARS-CoV-2 infection cases in pets, including a dog and three cats in São Paulo, Brazil.

**Materials and Methods:**

We invited COVID-19-positive pet owners to participate in the survey and obtained nasal, oropharyngeal, and rectal swab samples from their pets. These samples were placed in vials and subjected to a real-time quantitative polymerase chain reaction. In addition, the owners answered an epidemiological questionnaire, and the pets underwent clinical examination and monitoring.

**Results:**

Out of 49 sampled pets, 3/19 (15.8%) cats and 1/30 (3.3%) dogs tested positive, with wide variations in viral loads. Despite the limitations of size and non-randomized sampling, our results showed that cats are more susceptible than dogs to SARS-CoV-2 infection, presenting a cat: dog ratio of 4.8: 1. Only one cat presented mild and transitory respiratory symptoms.

**Conclusion:**

Although SARS-CoV-2 infection was detected in pets in the largest South American city and the COVID-19 epicenter at the time, these first detected pet cases displayed either none or mild clinical signs.

## Introduction

In December 2019, a novel strain of coronavirus, severe acute respiratory syndrome coronavirus 2 (SARS-CoV-2), was identified in Wuhan, China. This virus caused the coronavirus disease 2019 (COVID-19), which became a pandemic in early 2020, causing pneumonia and severe respiratory failure with high mortality among elderly, comorbid, and immunocompromised persons [1, 2]. SARS-CoV-2 belongs to the genus *Betacoronavirus*, which also includes the SARS-CoV and the Middle East respiratory syndrome coronavirus [3].

The virus is believed to have originated in bats as it shares 96% genomic similarity with bat coronavirus RaTG13 [1]. Another wildlife species that may have transmitted SARS-CoV-2 is the pangolin (*Manis javanica*), whose virus shares 85%–92% genomic similarity [3].

Since 2002, cats and ferrets have been reported to be susceptible to experimental infection with SARS-CoV, which uses the same angiotensin-converting enzyme II (ACE2) receptor as SARS-CoV-2 for human cell entry [4]. Despite a few amino acid differences in the binding sites between human and other mammal species, monkeys have identical regions and infections, indicating interspecies interaction [5]. Cats are more susceptible than dogs, and experimentally infected cats can transmit the disease to other cats through the air [6]. Moreover, other experimental infections have been reported in hamsters, ferrets, and monkeys [6]. Under experimental conditions, SARS-CoV-2 was previously detected in all three studied of cohort number 1 cat by plaque assay using oral and nasal swabs for up to 5 days post-infection (dpi). Viral RNA was detected in their oropharynx and nasal cavity for multiple dpi [7]. However, no SARS-CoV-2 infection was found in experimentally infected dogs. Another study reported detectable viral RNA in the organ tissues of 2/4 (50%) experimentally infected dogs after euthanasia at 4 dpi and was also transiently detected in the dog feces [6]. To the best of our knowledge, there are no studies which assessed SARS-CoV-2 reinfection in dogs and cats.

Human-to-animal transmission of SARS-CoV-2 was reported in captive tigers and lions at the New York Zoo, following respiratory signs in these animals and the detection of COVID-19 in the keepers [8]. Further, animal-to-human transmission was reported in mink farms when some workers were infected with strains with animal sequence signatures [9].

Although the SARS-CoV-2 infections reported in companion animals are generally asymptomatic and transitory, primarily due to close contact with infected owners [10], the role of companion animals in the life cycle of the virus remains unclear. This study aimed to survey SARS-CoV-2 infection in pets in São Paulo, Brazil, and detected the first pet cases of COVID-19 in a dog and three cats.

## Materials and Methods

### Ethics approval and Informed consent

This SARS-CoV-2 study was approved by the Ethics Committee for Animal Use (Protocol 4.879.280.420) of the Federal Rural University of Pernambuco. Verbal consent was obtained from each human participant.

### Study period and location

The study was conducted from October 2020 to July 2021 in São Paulo city, Brazil. São Paulo city has been ranked as the most populated and the second largest Gross Domestic Product in Latina America with around 12.4 million inhabitants, a high Human Development Index (0.805), and average temperatures of humid subtropical climate from 19°C (winter) to 25°C (summer).

The study herein was a longitudinal prospective analysis of dogs and cats in São Paulo city (23° 32′ 56” S; 46° 38′ 20″ W), whose owners were self-isolated at home due to confirmed SARS-CoV-2 diagnosis by real-time quantitative polymerase chain reaction (RT-qPCR).

### Sampling

COVID-19-positive pet owners voluntarily contacted us by phone or e-mail, and during the appointments, consents were signed. Nasopharyngeal and rectal swab samples were obtained from the dogs and cats by a certified veterinarian, placed in media tubes at 4°C, and sent for SARS-CoV-2 RT-qPCR testing to TECSA, a major accredited animal diagnostic laboratory located in Belo Horizonte, Southeast Brazil. After vortexing the transportation tubes with the swab samples for 30 s, 500 μL was used for RNA extraction using a commercial kit (Maxwell RSC simplyRNA Tissue Kit, Promega Co., Madison, WI, USA) on an automated platform (Maxwell RSC 48, Promega Co., Madison, WI, USA), following the manufacturer’s protocol. We conducted (GoTaq 1-Step RT-Promega Co., Madison, WI, USA) SARS-CoV-2 RT-qPCR (2019-nCoV, Integrated DNA Technologies – IDT, Coralville, IA, USA) targeting two SARS-CoV-2 regions for viral detection as previously described [8] using commercial RT-PCR kits and a commercial thermocycler (QuantStudio Block, Thermo Fisher, Waltham, MA, USA). The pre-cycles consisted of 50°C/15 min and 95°C/2 min, and 45 cycles of 95°C/30 s and 55°C/30 s. Test results with a cycle threshold (Ct) of <40 with amplification of at least two SARS-CoV-2 genes were considered positive. The amplification of a single target was considered inconclusive. All samples were tested in duplicate, and SARS-CoV-2 RNA was simultaneously quantified. The standard curve was plotted using 5-dilution points of a commercial SARS-CoV-2 sequence (2019-nCoV, IDT) as the positive control. Feline and canine b-actin were used as internal control genes, respectively.

## Results

In October 2021, we broadcasted and advertised a survey of pet owners who had recently tested positive (no longer than 7 days) in São Paulo and five other Brazilian capital states, including Belo Horizonte, Campo Grande, Cuiaba, Curitiba, and Recife. After being contacted by the SARS-CoV-2-positive owners, we immediately arranged for visits. Out of 49 sampled pets, 3/19 (15.8%) cats (SP-12F, SP-25F, and SP-28F) and 1/30 (3.3%) dogs (SP-17C) tested positive. In all the cases, more than one person had recently tested positive for SARS-CoV-2, and the pets had no outdoor access.

Of the three positive cats, SP-28F had the highest viral load (target N1: CT 25.15; copies: 1,640.31 RNA/μL; target N2: CT 26.25; copies: 1,063.52 RNA/μL) compared to SP-25F (target N1: CT 28.17; copies: 460.25 RNA/μL; target N2: CT 26.94; copies: 1006.30 RNA/μL) and SP-12F (target N1: CT 28.99; copies:) 313.77 RNA/μL; target N2: CT29.15; copies: 423.27 RNA/μL). While both samples of the COVID-19-positive dog SP-17C were positive, the viral load in the oropharyngeal (target N1: CT 33.25; copies: 6.14 RNA/μL; target N2: CT 30.78; copies: 23.35 RNA/μL) swab was higher than the rectal (target N1: CT 35.90; copies: 1.09 RNA/μL; target N2: CT 35.45; copies: 1.93 RNA/μL) one.

Only the cat SP-12F displayed non-specific clinical signs, including sneezing, nasal secretion, and itching a few days before sampling, which may or may not be related to the SARS-CoV-2 infection. All the pets were remotely monitored after testing, and their owners reported no health changes.

## Discussion

The SARS-CoV-2-positive pets belonging to COVID-19-positive owners in São Paulo indicate that human-to-animal transmission is possible, as previously observed [11]. Despite the biased starting algorithm of the positive owners applied in this study, no evidence of animal-human transmission was observed, as previously suggested [11, 12]. However, the methodology used here indicates that, even with positive owners, the frequency of infection may be as low as 15.8% (3/19) in cats and 3.3% (1/30) in dogs. Unsurprisingly, the infected cats and dogs were either asymptomatic or displayed transient symptoms, as shown in the early clinical case reports worldwide [10, 13, 14]. A recent study in Northeast Brazil showed that 0/16 dogs and 2/15 (13.3%) cats without clinical signs were considered positive for SARS-CoV-2 due to close contact with positive owners [13].

Despite the limitations, such as sample size and non-randomized sampling, this study has corroborated that cats may be more susceptible than dogs to SARS-CoV-2 infection. The cat: dog ratio was 4.8: 1, probably associated with ACE-2 receptor occurrence in cats and naturally occurring reported cases worldwide [15]. Nonetheless, the tested cats were either asymptomatic or mildly affected, with a single cat presenting only transitory respiratory signs, as opposed to that previously observed in a positive cat with dyspnea, diarrhea, and vomiting [16].

However, as we did not perform serological analysis in this study, the IgM/IgG antibody titers were not assessed in the infected dogs and cats. This limitation can be overcome by further investigating the antibody response. Although studies have shown that minks may transmit SARS-CoV-2 to their handlers, this study did not find evidence of pet-to-human transmission. However, that was not the aim of our study, and further studies should focus on the role of companion animals in SARS-CoV-2 transmission. Nonetheless, as the tested pets were infected by their owners, the precautions recommended for human-human transmission should be exercised, including self-isolation, use of a facial mask, and sanitization with alcohol after contact [17].

## Conclusion

Despite the limitations, this study showed no or mild clinical signs of SARS-CoV-2 infection in the pets of COVID-19-positive owners in São Paulo, the largest South American city and the pandemic epicenter at the time. We also showed that cats were more susceptible than dogs.

## Data Availability

The datasets generated and/or analyzed during the current study are available from the corresponding author on reasonable request.

## Authors’ Contributions

RGA, SCGL, AGBZ, GFS, IAVO, LSL, MAS, MCO, VFR: Collected the data. RGA, SCGL, OVC: Data analysis. RGA, DFB, OVC, CPB, APS, LBK, and AWB: Designed and performed the study and interpreted the data. All authors have drafted, revised, and approved the final manuscript.
